# Novel Stable Co_3_O_4_-SnO_2_ Heterojunction Electrocatalysts with Low Oxygen Evolution Potential

**DOI:** 10.3390/ma18081869

**Published:** 2025-04-19

**Authors:** Bingfeng Yan, Wen Liu, Youchen Sun, Meng Gao, Aqing Chen, Jun Zhang

**Affiliations:** New Energy Materials Research Center, College of Materials and Environmental Engineering, Hangzhou Dianzi University, Hangzhou 310018, China; yanbingfeng97@163.com (B.Y.); 221200048@hdu.edu.cn (W.L.); 18234165653@163.com (Y.S.); 15981996728@163.com (M.G.); zhangj@hdu.edu.cn (J.Z.)

**Keywords:** oxygen evolution potential, water electrolysis, node catalyst, SnO_2_ catalyst

## Abstract

Proton exchange membrane (PEM) water electrolysis offers a sustainable route for hydrogen production, yet the reliance on costly noble metal-based anodes hinders scalability. Tin dioxide (SnO_2_) emerges as a promising alternative due to its acid stability, but its high oxygen evolution potential (OEP) limits practical application in hydrogen production via water electrolysis. Here, we address this challenge by incorporating cobalt (Co) into SnO_2_ to create a heterojunction electrocatalyst. The optimized Co_3_O_4_-SnO_2_ heterojunction catalyst with a tin-to-cobalt mass ratio of 3:1 exhibits a significantly reduced OEP (1.6 V vs. RHE) and an overpotential of 186 mV at 10 mA cm^−2^ in acidic media, outperforming undoped SnO_2_. Stability tests reveal a lifespan exceeding 24 h at 100 mA cm^−2^, a threefold improvement over pure SnO_2_. This work underscores the potential of the Co_3_O_4_-SnO_2_ heterojunction electrocatalyst as a cost-effective, durable anode catalyst for PEM electrolyzers.

## 1. Introduction

Hydrogen, as a carbon-neutral energy carrier, is an efficient and clean energy source with no pollution and pivotal to global efforts in decarbonizing industries and transportation. Among technologies for green hydrogen production, proton exchange membrane water electrolysis (PEMWE) stands out for its high efficiency, rapid response, and compatibility with renewable energy sources [1,2]. However, the widespread adoption of PEMWE is hindered by its reliance on noble metal-based catalysts [3,4], such as iridium oxide (IrO_2_) and ruthenium oxide (RuO_2_) [5], for the anodic oxygen evolution reaction (OER). These materials are not only scarce and costly but also subject to supply chain vulnerabilities, underscoring the urgent need to develop affordable, high-performance alternatives. Consequently, research has focused on identifying non-noble metal oxide catalysts [6]. Tin dioxide (SnO_2_), with its exceptional stability in acidic environments, has emerged as a candidate for OER catalysis [7,8,9,10,11]. Yet, its high oxygen evolution potential (OEP ~2.0 V vs. RHE) severely limits practical application, necessitating innovative strategies to enhance its catalytic activity while preserving durability [12].

Recent advancements in SnO_2_ modification highlight both progress and unresolved challenges. While doping SnO_2_ with precious metals like Ir and Ru has improved catalytic performance [13,14]. For example, Xu achieved a lower OEP (1.7 V) by incorporating Ru into SnO_2_ [15]. But this approach escalated material costs, negating SnO_2_’s economic advantage. By 2022, attention shifted to non-noble metals. Joshi reported that copper (Cu)-doped SnO_2_ exhibited enhanced durability in acidic media [16]. Unfortunately, these studies collectively underscore a critical gap: existing research has not yet addressed the dual challenge of achieving low OEP and prolonged stability in SnO_2_ without relying on noble metals or compromising acid compatibility To bridge this gap, we propose a functionalized SnO_2_ electrocatalyst through cobalt doping. Cobalt, as an abundant and cost-effective non-precious metal, exhibits excellent catalytic activity under alkaline conditions and has gained attention as a promising substitute for precious metals [17]. In recent years, it has been widely used in the field of catalysts and is considered to be a promising substitute for noble metals [18,19]. Cobalt (Co) has gained traction as a cost-effective OER catalyst in alkaline environments. Li revealed that Co_3_O_4_ nanomeshes significantly accelerated OER kinetics in alkaline conditions [20]. Cobalt’s versatility in catalytic applications, rooted in its ability to adopt multiple oxidation states, suggests its potential to enhance OER activity of SnO_2_. Therefore, we choose to introduce the Co element into SnO_2_ as a modifier in order to improve the catalytic activity of the SnO_2_ catalyst through the high catalytic activity of Co in an alkaline environment, and to solve the problem of the poor stability of Co under acidic conditions with the help of SnO_2_ acid resistance.

However, integrating Co into SnO_2_ for acidic OER requires overcoming inherent material incompatibilities. Unlike prior studies that focused on single-phase doping, we chose to construct the SnO_2_-Co_3_O_4_ heterojunction structure. The core advantage of heterojunction structure OER catalysts lies in the multiple optimization of activity, stability, and conductivity through interfacial electronic reconstitution, component synergy, and structural design. Wu’s team [21] prepared NiFe-LDH/Ni_4_Mo alloy heterojunctions, and through the “oxygen pump” strategy, the Ni_4_Mo alloy provided oxidation intermediates and electrons, promoted lattice oxygen regeneration, and significantly reduced the overpotential (192.5 mV@10 mA cm^−2^). Coincidentally, Meng [22] and his team prepared a medium-entropy spinel oxide FeNiMnO_4_/CeO_2_ heterojunction electrocatalyst to form a built-in electric field to improve the conductivity, and CeO_2_ acted as an “electron pump” to regulate the center of the d-band close to the Fermi level, enhancing the adsorption capacity of the key intermediate (*OOH), and achieving an overpotential of 241 mV under alkaline conditions. Our approach leverages the synergistic interaction between Co_3_O_4_ and SnO_2_ to create a heterojunction structure that not only capitalizes on the catalytic activity of Co_3_O_4_ but also preserves the acid resistance of SnO_2_, addressing both performance and stability limitations.

In this work, Co_3_O_4_-SnO_2_ heterojunction catalysts were synthesized via a scalable high-temperature calcination method, and we systematically evaluate their structural, electrochemical, and stability properties. Structural characterization confirms the coexistence of SnO_2_ (rutile phase) and Co_3_O_4_ (spinel phase). Electrochemical testing reveals that the optimal Co_3_O_4_-SnO_2_ heterojunction catalyst achieves an OEP of 1.6 V and an overpotential of 186 mV at 10 mA cm^−2^ in 0.5 M H_2_SO_4_, outperforming undoped SnO_2_ and most non-precious metal catalysts. Stability tests further demonstrate uninterrupted operation for over 24 h at 100 mA cm^−2^, a three-fold improvement over pure SnO_2_. Mechanistic studies attribute this enhancement to the Co_3_O_4_-SnO_2_ heterojunction structure, which may optimize the carrier transport mechanism and significantly improve the conductivity and catalytic performance of the catalyst.

## 2. Experimental Methods

### 2.1. Catalyst Preparation

**Figure 1:**
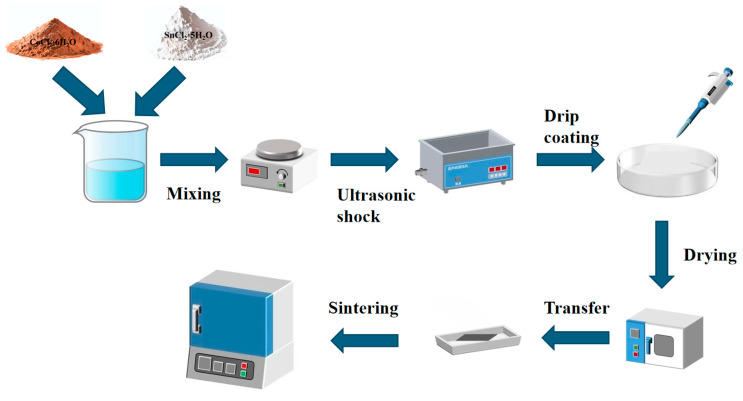
Sample preparation flow chart.

The precursors were prepared by dissolving SnCl_4_·5H_2_O and CoCl_2_·6H_2_O in 10 mL of absolute ethanol, stirring at 800 r/min. To enhance oxide film conductivity, 2 wt% SbCl_3_ was added to the solution. High-purity titanium plates (20 mm × 10 mm × 0.5 mm) were used as substrates. Electrodes were prepared via high-temperature calcination, following immersion in the precursor solution and brushing. The titanium plate surfaces were left to stand for two minutes and then dried at 150 °C for five minutes. Calcination at 450 °C in a muffle furnace for 10 min was repeated 10 times to ensure a uniform catalyst coating. The samples underwent final annealing at 450 °C for 2 h before cooling. Co_3_O_4_ and SnO_2_ monometal oxides were also prepared as controls using the above method. These catalyst samples are shown in Table 1, and the sample preparation flow chart is shown in Figure 1.

### 2.2. Characterization of Catalysts

Crystal structures were characterized using X-ray diffraction (XRD) with a MiniFlex600 diffractometer (XRD; MiniFlex600, Rigaku, Japan) equipped with CuKα radiation at 40 kV and 140 mA, scanning at 10° min^−1^ over 2θ angles from 10° to 80°. The surface elements’ chemical state and elemental composition were analyzed using an X-ray photoelectron spectrometer (XPS; Thermo Fisher Scienticfic K-Alpha; Nexsa; Thermo, Waltham, MA, USA), with all peak locations calibrated against the C1s peak at 284.8 eV. Surface morphology was analyzed via field emission scanning electron microscopy (FE-SEM, S-4200, Hitachi, Tokyo, Japen) at 2.0 kV, where the working distance was 4 mm, magnification was 5000×, and detector mode was SE. Transmission electron microscopy (TEM, FEI Talos F200X, Thermo Fisher Scientific, Waltham, MA, USA) provided further insights into catalyst morphology at 200 kV acceleration.

Electrochemical tests employed an LK2010 potentiostat in a standard three-electrode setup, with a platinum sheet as the counter electrode, a mercurous sulfate reference electrode, and 0.5 M H_2_SO_4_ electrolyte. Measured potentials were converted to reversible hydrogen electrode (RHE) potentials using the Nernst equation:

E_RHE_ = E_1_ + 0.0592pH + 0.656 (1)

Linear sweep voltammetry (LSV) curves for OER were obtained at 50 mV/s over a 0–2.5 V range, corrected with 95% iR compensation. Double-layer capacitance (Cdl) was determined via cyclic voltammetry (CV) tests at 20–120 mV/s within the non-Faradaic region. Chronopotential stability tests were conducted at a current density of 100 mA cm^−2^.

## 3. Results

### 3.1. Catalyst Structural Analysis

**Figure 2:**
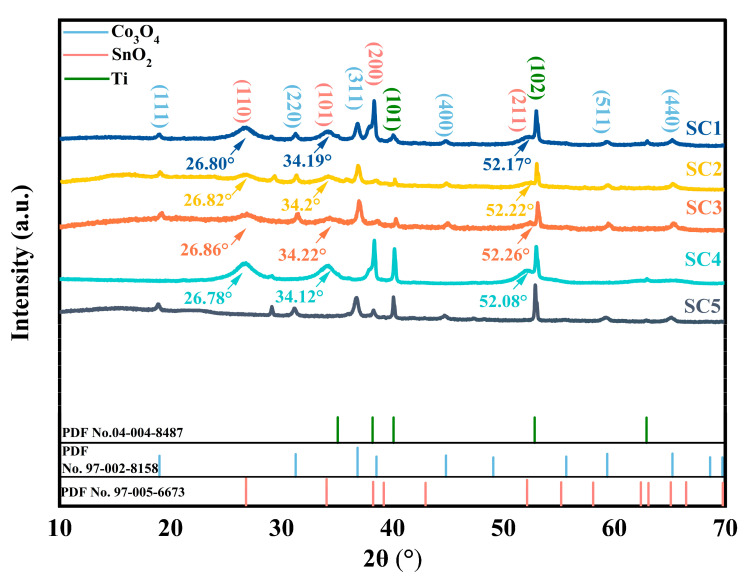
XRD patterns of SC1–SC5 catalysts and Ti, Co_3_O_4_, and SnO_2_ standard PDF card diagrams.

To determine the phase composition of the sample, XRD analysis was conducted. Figure 2 shows the XRD pattern of the five groups of catalysts. It was determined that the six diffraction peaks observed at 18.891°, 31.159°, 36.711°, 44.709°, 59.190° and 65.092° in the SC1, SC2, and SC3 catalysts could be attributed to the crystal planes (111), (220), (311), (400), (511), and (440) of Co_3_O_4_, by comparison with standard PDF cards (PDF No. 97-002-8158). Furthermore, the presence of four diffraction peaks was detected at 26.888°, 34.137°, 38.392°, 52.124°, and 55.541°, which correspond to the crystal planes (110), (101), (200), and (211) of SnO_2_, respectively, as identified by comparison with the standard PDF card (PDF No. 97-005-6673). In addition, the (101) and (102) crystal planes associated with Ti atoms were found at 40.06° and 52.9° (PDF No. 04-004-8487).

The XRD patterns of the SC1, SC2, and SC3 catalysts revealed the presence of distinct diffraction peaks corresponding to SnO_2_ and Co_3_O_4_, which were in close agreement with those (SC4 and SC5) of the respective control samples. This indicated that the Co atom was successfully introduced. Among them, the diffraction peaks of (101), (110), and (211) crystal planes of SC1, SC2, and SC3 catalyst samples had a peak shift compared with SC4 samples, and the SC1–SC3 catalysts had varying degrees of high angular shifts of about 0.1°. According to the Bragg equation, the crystal plane of SnO_2_ was shrunk. The intensity of the diffraction peaks of (101), (110), and (200) crystal planes gradually decreased with the increase in Co molar ratio. At the same time, the diffraction peaks of the (311) and (440) crystal planes of these three groups of samples also shifted compared with the SC5 samples, and the intensity of the diffraction peaks gradually increased with the increase in the Co molar ratio. No mixed phase of Co_3_O_4_ and SnO_2_ was observed in the XRD pattern. SnO_2_ is known to crystallize in a rutile structure, characterized by lattice parameters of a = 0.47373 nm and c = 0.31864 nm. Conversely, the Co_3_O_4_ phase adopts a spinel structure, characterized by a lattice parameter of a = 0.8084 nm. The significant difference in lattice parameters between the two phases suggests that they are unlikely to form a continuous solid solution, in accordance with the Hume–Rothery rules [23]. Moreover, there was an absence of any additional diffraction peaks distinct from those of SnO_2_ and Co_3_O_4_ in the XRD patterns of those catalysts. Those observations suggest that these catalysts do not produce alloying compounds, but are heterojunctions.

**Figure 3:**
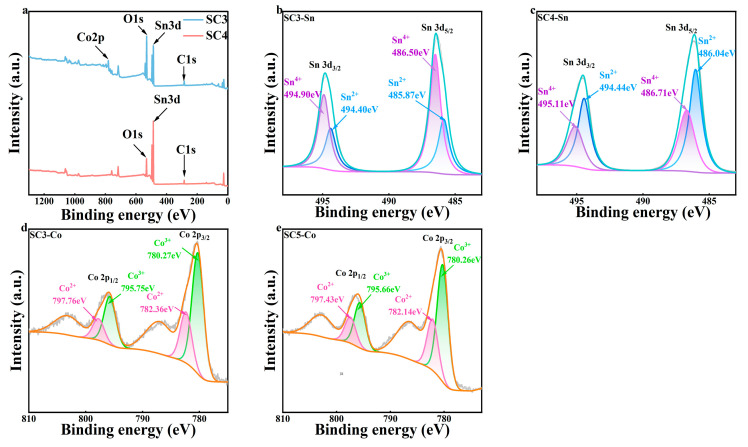
(**a**) Full XPS patterns of SC3 and SC4, (**b**) Sn 3d of SC3, (**c**) Sn 3d of SC4, (**d**) Co 2p of SC3, (**e**) Co 2p of SC5.

According to the accepted principle, the heterostructures can also affect the surface chemical state of metal oxides compared to the original oxides [24]. Therefore, XPS is used to analyze the chemical state of these samples, as shown in Figure 3. Figure 3a shows the full XPS spectrum of SC3 and SC4, with characteristic peaks indicating that the Co element was successfully incorporated into the SC3 catalyst sample, unlike SC4.

Figure 3b is the resolution of the Sn3d spectrum with SC3. The binding energies located at 494.53 eV and 486.18 eV are attributed to Sn 3d_5/2_ and Sn 3d_3/2_. The fitted peaks at 486.50 eV and 494.90 eV belong to Sn4^+^, and the fitted peaks at 485.87 eV and 494.40 eV belong to Sn^2+^. Figure 3c is the resolution of the Sn3d spectrum with SC4. The binding energies located at 494.58 eV and 486.28 eV are attributed to Sn 3d_5/2_ and Sn 3d_3/2_. The fitted peaks at 486.71 eV and 495.11 eV belong to Sn4^+^, and the fitted peaks at 486.04 eV and 494.44 eV belong to Sn^2+^ [25,26]. When Co atoms are introduced into the sample, the Sn 3d_5/2_ and Sn 3d_3/2_ peaks are negatively shifted. This result may be attributed to the greater electronegativity of Sn (1.96) than that of Co (1.88). Due to the greater electronegativity of Sn, the Sn 3d electrons are closer to the nucleus, resulting in a decrease in binding energy [27].

Figure 3d shows the high-resolution XPS spectrum of the Co of SC3. There are two characteristic peaks, of Co 2p_3/2_ (780.48 eV) and Co 2p_1/2_ (795.98 eV); this result is similar to that found in the literature [28,29]. The gap between two spin-orbital twin peaks is 15.50 eV, which proves the coexistence of Co^3+^ and Co^2+^. Diffraction peaks of 782.36 eV and 797.76 eV can be fitted to Co^3+^, while those at 780.27 eV and 795.75 eV are the positions of Co^2+^. Figure 3e shows the high-resolution XPS spectrum of the Co of SC5. There are two characteristic peaks, of Co2p_3/2_ (780.53 eV) and Co2p_1/2_ (796.03 eV), and the gap between two spin-orbital twin peaks is 15.50 eV, which proves the coexistence of Co^3+^ and Co^2+^. Diffraction peaks of 782.14 eV and 797.43 eV can be fitted to Co^3+^, while those at 780.26 eV and 795.66 eV are the positions of Co^2+^. All peaks in SC3 have a positive shift relative to SC5; this is due to the presence of tin oxide and stannous oxide [30].

**Figure 4:**
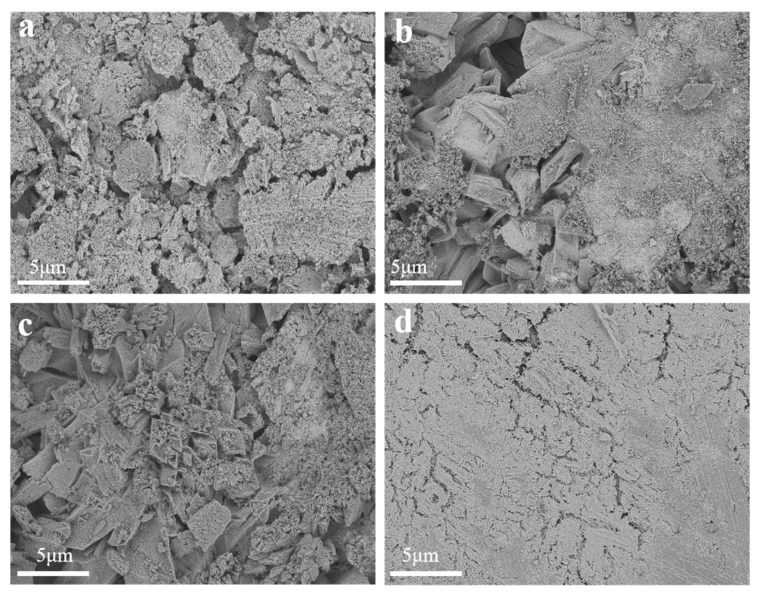
(**a**) FE-SEM pattern of SC1 catalyst sample, (**b**) FE-SEM pattern of SC2 catalyst sample, (**c**) FE-SEM pattern of SC3 catalyst sample, (**d**) FE-SEM pattern of SC4 catalyst sample.

In order to describe the further morphologies of the sample, FE-SEM was employed to examine the surface of the composite catalysts. Figure 4a, Figure 4b, and Figure 4c, respectively, show morphologies of SC1, SC2, and SC3; it revealed that the surface of the SC1, SC2, and SC3 catalysts constitutes Co_3_O_4_ and SnO_2_ particles. Figure 4d presents surface FE-SEM images about the SC4 catalyst sample without the Co element, where granular cracks and a sparse distribution of surface pores characterize the catalyst surface. Upon the incorporation of Co, it was observed that the surface topography of the catalyst sample significantly changed. It is not difficult to see that compared with SC4, the surface pores of the SC1, SC2, and SC3 catalyst samples are increased and massive nanostructures appear. With the increase in Co concentration, the surface bulk nanostructures also increase. Concurrently, a discernible enhancement occurs in surface porosity and crystallinity. The abundance of surface pores exhibits a complex interconnection with the catalytic activity of the catalyst. In fact, the higher the density of these pores, the more active sites there are, thus providing more attachment sites for the reaction of water molecules to decompose and accelerating the anodizing process [31]. This morphological evolution, as evidenced by the FE-SEM analysis, underscores the significance of Co incorporation in modulating the surface properties and consequently the catalytic performance of the heterostructured catalyst.

**Figure 5:**
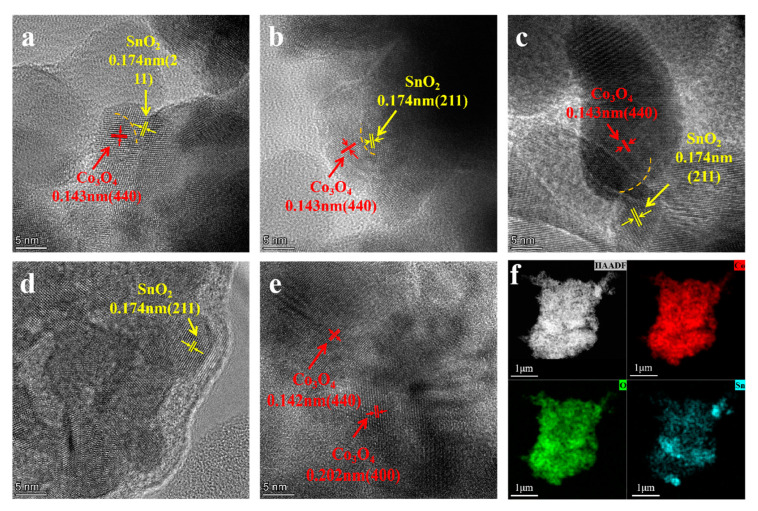
(**a**) TEM pattern of SC1 catalyst sample, (**b**) TEM pattern of SC2 catalyst sample, (**c**) TEM pattern of SC3 catalyst sample, (**d**) TEM pattern of SC4 catalyst sample, (**e**) TEM pattern of SC5 catalyst sample, (**f**) mapping of SC3 catalyst sample.

Figure 5a, Figure 5b, and Figure 5c show TEM patterns of the sample surfaces of SC1, SC2, and SC3 catalysts, respectively. The lattice fringes of 0.174 nm and 0.143 nm are clearly shown in the figure, respectively, belonging to the (211) plane of SnO_2_ (these lattice fringes can also be found in SC4, Figure 5d) and the (440) plane of Co₃O₄ (these lattice fringes can also be found in SC5, Figure 5e), which corresponds to the results of XRD (Figure 2). Phase interfaces (marked with orange dotted lines) were observed in the TEM patterns of all three groups of samples, demonstrating the formation of a compact Co_3_O_4_-SnO_2_ heterojunction structure. Furthermore, high-angle annular dark-field (HAADF) imaging was used to visualize the distribution of Co, Sn, and O elements within the composite structure. In Figure 5f, the HAADF images clearly demonstrate that all elements are uniformly distributed throughout the entire structure, indicating a homogeneous composition and structural integrity of the heterostructured catalyst. The red color is Co, blue color is Sn, and green color represents O. This uniform elemental distribution is crucial for the synergistic catalytic activity of the composite, as it ensures that each component can effectively contribute to the catalytic process.

### 3.2. Electrochemical Performance Analysis of Catalysts

**Figure 6:**
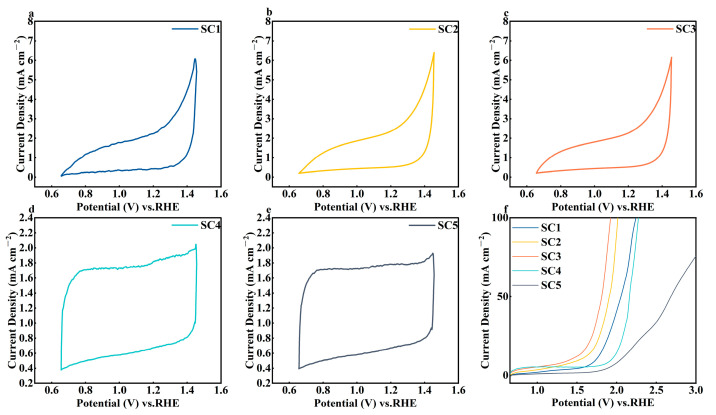
(**a**) SC1 CV curve, (**b**) SC2 CV curve, (**c**) SC3 CV curve, (**d**) SC4 CV curve, (**e**) SC5 CV curve, (**f**) SC1-5 LSV curve.

The electrochemical performance of several groups of catalyst samples with different cobalt concentrations was evaluated using 0.5 mol/L H_2_SO_4_ as the electrolyte solution. Figure 6a–e show the CV curves for each catalyst obtained by cyclic scanning voltammetry at a scan speed of 50mV/s. It is not difficult to see from the figure that the SC1, SC2, and SC3 catalyst samples all had obvious oxidation reactions in the range of 1.2 V~1.4 V, and the potential of the oxidation peaks of the three groups of catalyst samples was essentially the same, while the SC4 catalyst without the Co element and the SC5 catalyst without the Sn element did not have clear oxidation reactions in the same voltage range. This phenomenon proves that the formation of the heterojunction can effectively improve the activity of the catalyst.

Figure 6f shows the LSV curves for the five groups of catalyst samples. Compared with the oxygen evolution potential of SC4 samples of about 2.0 V, the oxygen evolution potential of SC1, SC2, and SC3 catalysts was significantly reduced. Moreover, when the concentration of Co increases, the oxygen evolution potential shows a further decreasing trend. The oxygen evolution potential of the SC3 catalyst with the largest Co concentration is about 1.6 V, which is a huge decrease compared with SC4.

**Figure 7:**
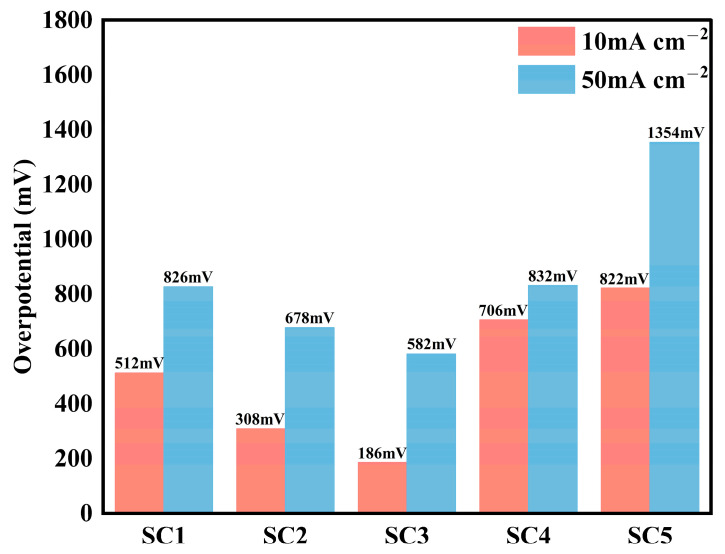
Overpotential of SC1, SC2, SC3, SC4, and SC5 catalyst samples.

The acidic OER activity of the catalyst was determined by calculating the overpotential at a current density of 10 mA cm^−2^ (Figure 7). Comparative analysis showed that the SC4 catalyst had an overpotential of 706 mV at 10 mA cm^−2^ without Co. It is worth noting that the overpotential of the three groups of heterojunction catalysts with different Co concentrations was significantly reduced. The best-performing SC3 catalyst exhibited an overpotential of 186 mV at 10 mA cm^−2^.

**Figure 8:**
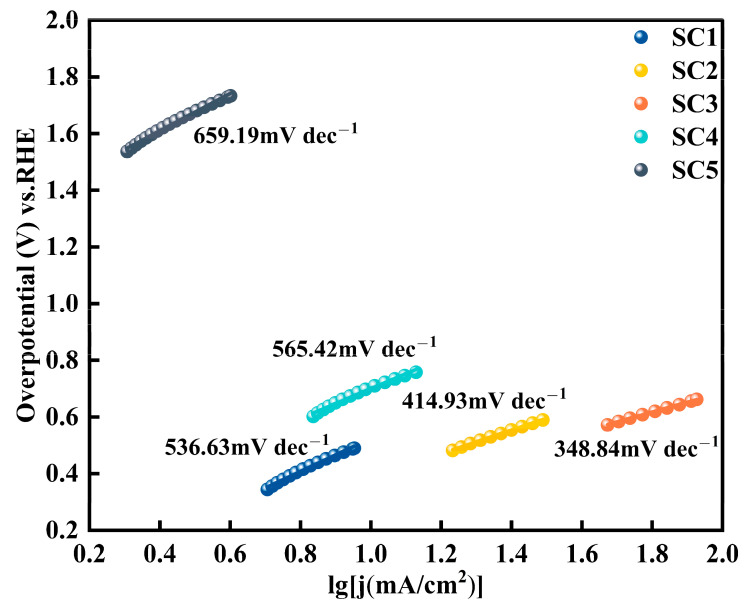
Tafel slopes of catalyst samples.

The Tafel slope is an important parameter used to evaluate the reaction speed of the catalyst in the water splitting reaction, and its value is obtained by fitting the LSV polarization curve by Equation (1):

η = a + b logj(2)

where η is the overpotential, a means constant, b is the Tafel slope, and j is the measured current density. An elevated η value indicates a slower response, and conversely, a smaller value indicates a faster response. As shown in Figure 8, the Tafel slope of the SC4 catalyst without Co is 565.42 mV dec^−1^. The Tafel slopes of SC1, SC2, and SC3 catalysts decreased after the addition of different concentrations of Co, and the Tafel slope of the SC3 catalyst was the lowest, at only 348.84 mV dec^−1^. These results indicate that the formation of the Co_3_O_4_-SnO_2_ heterojunction optimizes the reaction kinetics of the catalyst, makes it more catalytically active, and accelerates the progress of the water splitting reaction.

**Figure 9:**
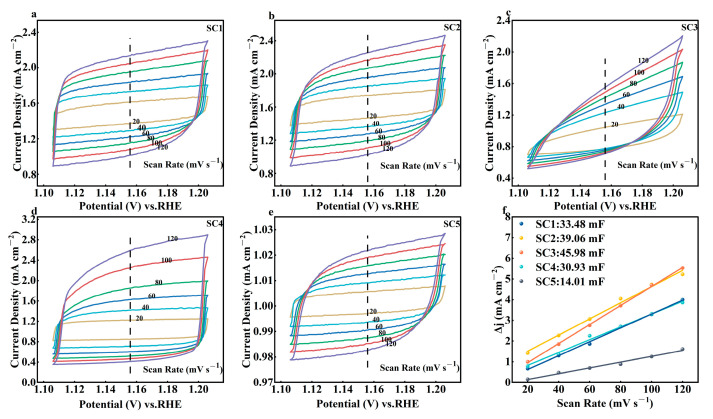
(**a**–**e**) Electric two-layer scan curve of SC1~ SC5, (**f**) Cdl of catalyst samples.

To obtain the electrochemical surface area (ECSA) of the catalyst in order to assess the active site, the electrical Cdl of the catalyst was calculated from CV curves of the sample at different scan velocities in the non-Rady current region. As shown in Figure 9, the scanning speed is set to 20~120 mV s^−1^. After calculation, the results show that the Cdl values (SC1: 33.48 mF, SC2: 39.06 mF, SC3: 45.98 mF) of the three groups of catalyst samples forming the heterojunction are increased compared with the 30.93 mF of SC4.

The ESCA value can be calculated from the Cdl value. According to Equation (2),

ECSA = Cdl/Cs(3)

**Figure 10:**
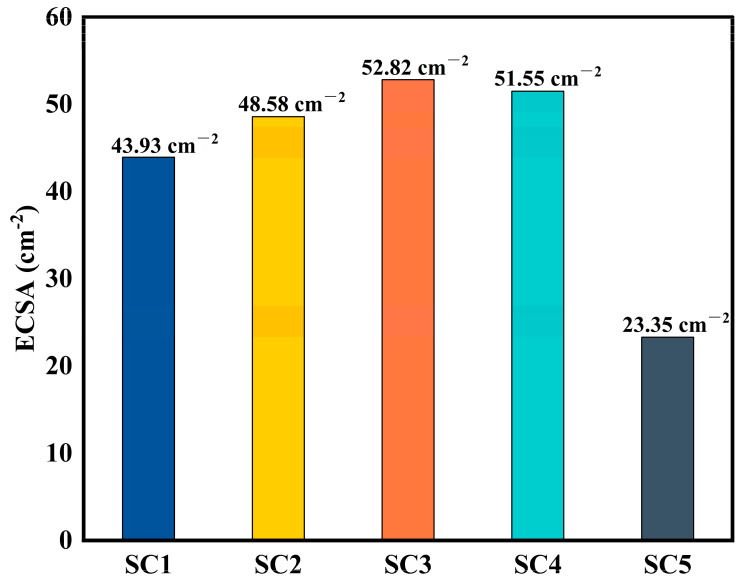
ECSA of catalyst samples.

Cs is the specific capacitance. The counter electrode used in the experiment is a platinum sheet with a specific capacitance of 60F cm^−2^. Due to the different loading amounts of the catalyst on the SC1–SC5 samples, we normalized the values of ECSA, as shown in Figure 10.

According to the ESCA of several groups of catalysts, it was found that compared with the 51.55 cm^−2^ of SC4, the ESCA values of SC1 (43.93 cm^−2^), SC2 (48.58 cm^−2^), and SC3 (52.82 cm^−2^) catalysts with heterojunction formation increased gradually, and they showed a trend of increasing ESCA with the increase in Co concentration. The formation of this surface heterojunction increases the actual electrochemically active area of the catalyst surface, providing more active sites and increasing the reaction rate, which also matches the results of the Tafel slope.

**Figure 11:**
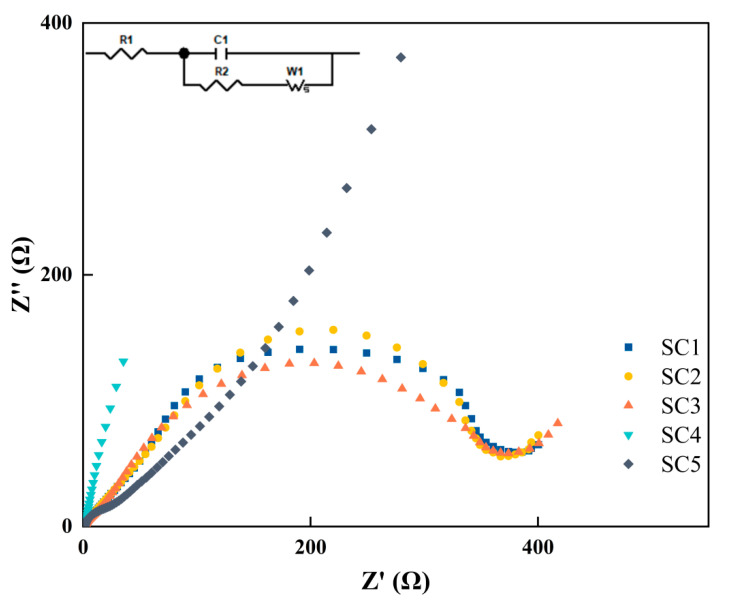
EIS of catalyst samples.

The charge transfer resistance (Rct) in electrochemical impedance spectroscopy (EIS) reflects how easily electrons are transferred between the catalyst surface and the reactants. The smaller the RCT, the easier the electron transfer and the higher the catalytic activity. Figure 11 shows EIS plots of several groups of catalyst samples. The EIS values of the five groups of catalyst samples are as follows: SC1 is 381 Ω, SC2 is 365 Ω, SC3 is 352 Ω, SC4 is 1201 Ω, and SC5 is 4100 Ω. These results are attributed to the optimization of carrier transport at the two-dimensional heterojunction interface.

**Figure 12:**
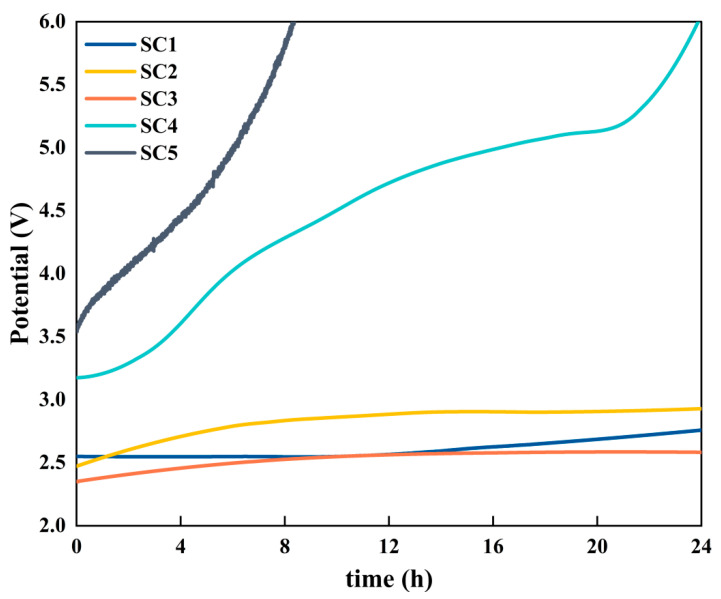
SC1–SC5 catalyst chronopotentionmetry test diagram.

In the evaluation of anode catalysts operating under acidic conditions, stability is a paramount criterion, complementing catalytic activity as a key performance indicator. As illustrated in Figure 12, the stability of the five groups of catalysts was compared and evaluated, and it was proven that the stability of the catalyst for the formation of the Co_3_O_4_-SnO_2_ heterostructure was much higher than that of the pure SnO_2_ catalyst and the Co_3_O_4_ catalyst. Stability was assessed at a current density of 100 mA cm^−2^ using a chronopotentiometric technique. The results show that the SC5 catalyst fails quickly under acidic conditions. The SC4 catalyst maintained stable electrolysis for about 4 h. After that, the electrolytic voltage gradually increased, and after about 8 h, the electrolytic voltage had risen by more than 1 V compared to the initial value. At the end of the 24 h test, the electrolytic voltage of the three groups of heterojunction catalyst samples, SC1, SC2, and SC3, had increased by about 0.3 V compared to the initial value, while that of the SC4 catalyst had increased by about 3 V. The SC4 catalyst surface has been completely passivated and has lost its catalytic activity. This observation highlights the enhanced stability conferred by the formation of the Co_3_O_4_-SnO_2_ heterojunction catalyst, although the performance of the catalyst still exhibits a decrease over time, underscoring the need for further optimization to improve long-term stability. This is due to the presence of a certain degree of non-stoichiometric ratio (SnO_2−x_) in the prepared SnO_2_ and the reaction of Equation (3) [32]:

SnO_(2−x)_ + H_2_O → SnO_(2−x)_(OH) + H^+^ + e^−^ SnO_(2−x)_(OH) → SnO_(2−x+y)_ + yH^+^ + ye^−^ (4)

This change in the surface of the anode increases the internal stress of the oxide layer, and the higher initial voltage accelerates this reaction rate, which eventually leads to rapid detachment of the oxide layer and failure of the catalyst. And when Co is added to form a heterojunction, the potential of the oxygen evolution reaction is reduced, thereby inhibiting the rapid shedding of the oxide layer, and the composite catalyst obtains better stability in acidic OER, while the service life is greatly extended. As shown in Table 2, the catalytic performance and stability of the SC3 catalyst are improved compared with other forms of SnO_2_ OER catalyst.

After the chronopotentionmetry test, in order to explore the structure and topography of the catalyst sample, the SEM of the sample surface was photographed again.

**Figure 13:**
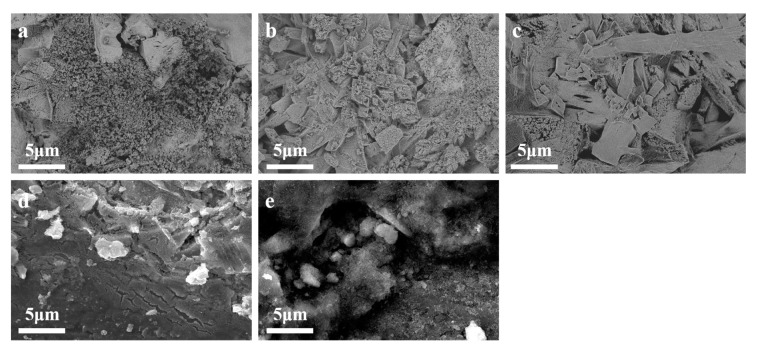
(**a**–**e**) SEM image of SC1–SC5 catalyst surfaces after chronopotentionmetry.

As can be seen from Figure 13, the surface of SC1, SC2, and SC3 composite catalysts had not changed significantly compared with before the chronopotentionmetry test, the surface massive nanostructures remained intact, and the catalytic performance had not changed greatly. However, the surface consistency of SC4 and SC5 was greatly damaged, and bare titanium plates appear in some areas of the figure (Figure 13d bottom left area and Figure 13e bottom right area), while the surface catalyst was passivated and peeled off. 

**Figure 14:**
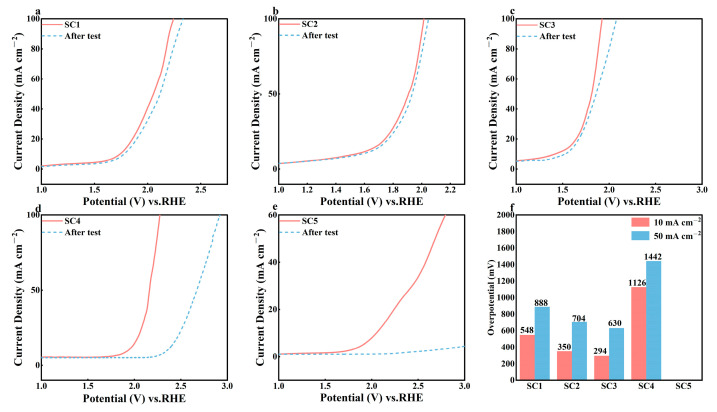
(**a**–**e**) LSV comparison chart before and after chronopotentionmetry of SC1–SC5 catalysts; (**f**) overpotential image of SC1–SC5 catalysts after chronopotentionmetry.

In order to explore the attenuation rate of catalyst performance, the LSV test was carried out on five groups of catalysts with the same test parameters, and a comparison chart of before and after the chronopotentionmetry test was drawn. As can be seen from Figure 14, the oxygen evolution potential of SC1, SC2, and SC3 composite catalysts increased significantly, and the reaction rate did not decrease significantly after the oxygen evolution reaction began compared with before the test. On the other hand, the oxygen evolution potential of the SC4 catalyst decreased significantly, from about 2.0 V before the test to about 2.4 V after the test. The SC5 catalyst failed directly, and there was no obvious oxygen evolution until 3.0 V. As shown in Figure 14f, the overpotential of SC1, SC2, and SC3 catalysts at 10 mA cm^−2^ and 50 mA cm^−2^ only increased slightly compared with that before the chronopotential test, especially the best SC3 catalyst, whose overpotential at 50 mA cm^−2^ only increased by 48 mV, with a performance loss of about 8%. However, the overpotential of the SC4 catalyst increased significantly after the test, and the catalytic performance decreased significantly. The SC5 catalyst could not detect the effective overpotential in the same test potential range, and the catalyst failed completely.

## 4. Conclusions

In summary, this study demonstrates that the Co_3_O_4_-SnO_2_ heterojunction reduces the OEP of SnO_2_ from 2.0 V to 1.6 V (vs. RHE), with an overpotential of 186 mV at 10 mA cm^−2^, rivaling noble metal benchmarks. Furthermore, it suggests that the Co_3_O_4_–SnO_2_ heterojunction enhances catalytic activity, reduces the oxygen evolution potential, and enhances durability (>24 h at 100 mA cm^−2^) by redistributing mechanical stress during OER. These findings provide insights into cost-effective OER catalyst design using non-noble metal elements, advancing alternatives to noble metal-based catalysts.

## Figures and Tables

**Table 1:** Catalyst electrodes with different Co contents.

Electrode Name	Cobalt–Tin Molar Ratio	Cobalt Loading per Monolith (mg cm^−2^)	Tin Loading per Monolith (mg cm^−2^)
SC1	1:5	2.7	10
SC2	1:4	3.4	10
SC3	1:3	4.5	10
SC4	No cobalt	0	10
SC5	No tin	10	0

**Table 2:** Comparison of catalytic performance and stability of several SnO_2_-based catalysts.

Catalyst Name	Overpotential at 10 mA cm^−2^	Stability at Acidic Condition
SC3	186 mV	24 h+
NiO-SnO_2_ [33]	320 mV	20 h+
CuO-SnO_2_ [34]	280 mV	10 h+
IrO_2_/Ti-SnO_2_ [35]	271 mV	5.5 h+

## Data Availability

The raw data supporting the conclusion of this article will be made available by the authors on request.

## List of Abbreviations

PEM	proton exchange membrane
OEP	oxygen evolution potential
PEMWE	proton exchange membrane water electrolysis
OER	oxygen evolution reaction
XRD	X-ray diffraction
XPS	X-ray photoelectron spectrometer
FE-SEM	field emission scanning electron microscopy
TEM	transmission electron microscopy
RHE	reversible hydrogen electrode
LSV	linear sweep voltammetry
Cdl	double-layer capacitance
CV	cyclic voltammetry
HAADF	high-angle annular dark-field
ECSA	electrochemical surface area
Rct	charge transfer resistance
EIS	electrochemical impedance spectroscopy
